# Contextual Determinants of Time to Surgery for Patients With Hip Fracture

**DOI:** 10.1001/jamanetworkopen.2023.47834

**Published:** 2023-12-15

**Authors:** Jessica M. Welch, Giselle I. Gomez, Maya Chatterjee, Lauren M. Shapiro, Arden M. Morris, Michael J. Gardner, Alex H. S. Sox-Harris, Laurence Baker, Jayme C. B. Koltsov, Tiffany Castillo, Nicholas Giori, Aaron Salyapongse, Robin N. Kamal

**Affiliations:** 1 VOICES Health Policy Research Center, Department of Orthopaedic Surgery, Stanford University, Redwood City, California; 2 Duke University School of Medicine, Durham, North Carolina; 3 Stanford University School of Medicine, Stanford, California; 4 Department of Human Development and Family Studies, Colorado State University, Fort Collins; 5 Department of Orthopaedic Surgery, University of California, San Francisco; 6 Department of Surgery, Stanford University, Stanford, California; 7 Department of Orthopaedic Surgery, Stanford University, Redwood City, California; 8 Veterans Affairs Palo Alto Health Care System, Palo Alto, California; 9 Department of Health Policy, Stanford University, Stanford, California; 10 Department of Orthopaedic Surgery, Santa Clara Valley Medical Center, San Jose, California; 11 Department of Orthopaedic Surgery, Veterans Affairs Palo Alto Health Care System, Palo Alto, California

## Abstract

**Importance:**

Surgery within 24 hours after a hip fracture improves patient morbidity and mortality, which has led some hospitals to launch quality improvement programs (eg, targeted resource management, documented protocols) to address delays. However, these programs have had mixed results in terms of decreased time to surgery (TTS), identifying an opportunity to improve the effectiveness of interventions.

**Objective:**

To identify the contextual determinants (site-specific barriers and facilitators) of TTS for patients with hip fracture across diverse hospitals.

**Design, Setting, and Participants:**

This qualitative mixed-methods study used an exploratory sequential design that comprised 2 phases. In phase 1, qualitative semistructured interviews were conducted with stakeholders involved in hip fracture care (orthopedic surgeons or residents, emergency medicine physicians, hospitalists, anesthesiologists, nurses, and clinical or support staff) at 4 hospitals with differing financial, operational, and educational structures. Interviews were completed between May and July 2021. In phase 2, a quantitative survey assessing contextual determinants of TTS within 24 hours for adult patients with hip fracture was completed by orthopedic surgeon leaders representing 23 diverse hospitals across the US between May and July 2022. Data analysis was performed in August 2022.

**Main Outcomes and Measures:**

Thematic analysis of the interviews identified themes of contextual determinants of TTS within 24 hours for patients with hip fracture. The emergent contextual determinants were then measured across multiple hospitals, and frequency and distribution were used to assess associations between determinants and various hospital characteristics (eg, setting, number of beds).

**Results:**

A total of 34 stakeholders were interviewed in phase 1, and 23 surveys were completed in phase 2. More than half of respondents in both phases were men (19 [56%] and 18 [78%], respectively). The following 4 themes of contextual determinants of TTS within 24 hours were identified: availability, care coordination, improvement climate, and incentive structure. Within these themes, the most commonly identified determinants across the various hospitals involved operating room availability, a formal comanagement system between orthopedics and medicine or geriatrics, the presence of a physician champion focused on timely surgery, and a program that facilitates improvement work.

**Conclusions and Relevance:**

In this study, contextual determinants of TTS within 24 hours for patients with hip fracture varied across hospital sites and could not be generalized across various hospital contexts because no 2 sites had identical profiles. As such, these findings suggest that guidance on strategies for improving TTS should be based on the contextual determinants unique to each hospital.

## Key Points

**Question:**

What are the contextual determinants of optimal time to surgery (TTS; <24 hours) among patients with hip fracture?

**Findings:**

In this qualitative study using mixed methods and including 34 semistructured interviews and 23 surveys, 4 themes emerged regarding contextual determinants of TTS: availability, care coordination, improvement climate, and incentive structure. Contextual determinants varied across hospital systems.

**Meaning:**

These findings suggest that improvement strategies for TTS for patients with hip fracture should be based on hospital-specific contextual determinants to achieve a higher likelihood of successfully preventing delays to surgery.

## Teaser

This qualitative study uses mixed methods to examine contextual determinants, such as site-specific barriers and facilitators, of time to surgery for patients with hip fracture across diverse hospitals.

## Introduction

Hip fractures are a common geriatric injury, with an annual incidence of 340 000 and an estimated cost of $34 000 to $54 000 per patient^1^ attributed to hospitalization and postsurgical care.^2,3^ Surgery within 24 hours reduces postoperative complications, length of hospitalization, and mortality^4,5,6,7^; however, only 22% of patients with hip fracture undergo surgery within 24 hours in the US, exposing more than 265 000 patients to unnecessary morbidity and mortality.^8^ Considering this evidence, the American Academy of Orthopaedic Surgeons (AAOS) released a clinical practice guideline in 2014, recommending surgical treatment of hip fracture within 24 to 48 hours for adults aged 65 years or older,^9^ and submitted this guideline to the US Centers for Medicare & Medicaid Services as a quality measure.

Delays in time to surgery (TTS) for patients with hip fracture persist, despite the AAOS guideline. Patient-level factors, such as Asian or Black race, male sex, lower income, or multiple comorbidities, have been associated with delayed TTS. Facility-level factors, including larger hospital size, annual surgical volume, and a medical optimization protocol, have also been associated with delays.^10,11,12^ To address delays in TTS, diverse strategies, including targeted resource management,^13,14,15^ comanagement programs between orthopedics and medicine or geriatrics,^16,17,18,19^ documented protocols,^20,21^ and financial incentives,^22,23,24^ have been implemented with varying levels of success.^17,19,25,26,27,28^

Challenges in implementing evidence-based improvement interventions include organizational knowledge, medical socialization (ie, medical decision-making based on knowledge from trusted peers or mentors rather than directly from peer-reviewed evidence), and patient and surgeon factors, among others.^29,30^ To be successful, evidence-based improvement interventions should be tailored to the context in which they will be implemented; however, there is currently no national-level guidance for how hospitals can improve TTS based on their own context. Although studies have identified factors associated with delayed TTS, less is known about the contextual determinants (ie, site-specific barriers and facilitators) that influence TTS for individual hospitals. We aimed to identify contextual determinants of achieving TTS of less than 24 hours for patients with hip fracture and to assess the variability of these determinants between hospitals.

## Methods

### Study Design

This mixed-methods qualitative study was conducted in accordance with ethical principles and guidelines outlined by the Declaration of Helsinki.^31^ The Stanford University Institutional Review Board approved the study protocol. All participants provided verbal or written agreement to participate prior to study enrollment. This study was designed and reported in accordance with the Consolidated Criteria for Reporting Qualitative Research (COREQ) checklist.

We used an exploratory sequential mixed-methods study design that comprised 2 phases (Figure), with integration of the qualitative and quantitative findings to identify contextual determinants of TTS for adult patients with hip fracture in the US.^32^ This method involves knowledge generation through a qualitative phase to inform development of the subsequent quantitative phase, and it allows for integration of results to identify common themes that would be otherwise challenging to extrapolate using either method in isolation. First, we used a qualitative descriptive design and conducted semistructured interviews with stakeholders involved in hip fracture care across 4 affiliated hospitals, each with distinct financial (private vs public medical centers), operational (level I vs non–level I trauma centers), and educational structures (teaching vs nonteaching hospitals). After thematic analysis, we used the emergent themes to develop a quantitative assessment, which we distributed to a nationwide sample of orthopedic surgeons.^33,34^

**Figure.**
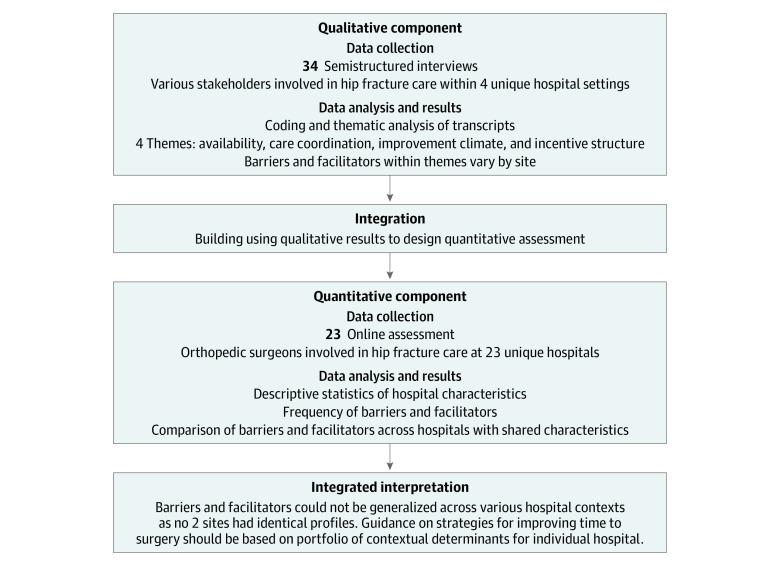
Exploratory Sequential Mixed-Methods Process

### Participants

Semistructured interview participants were recruited through purposive sampling, wherein the chief of orthopedic surgery at each hospital contacted stakeholders across different departments (eg, operating room [OR] charge nurses, quality improvement [QI] specialists, orthopedic surgeons, hospitalists or geriatricians, and anesthesiologists) who could provide insight into aspects of care that affect TTS. The assessment was distributed via email to orthopedic surgeons involved in hip fracture care representing hospitals across the US that are members of the Surgical Wait Time for Fracture Treatment (SWIFT) Initiative, a multicenter collaboration working to improve TTS for patients with hip fracture. The SWIFT sites were purposively sampled to ensure diverse representation of geographic location, patient demographics, and payer type variation.

### Qualitative Data Collection and Thematic Analysis

We developed an interview guide (eAppendix 1 in Supplement 1) based on both the Consolidated Framework for Implementation Research (CFIR) and the Theoretical Domains Framework (TDF). The CFIR is commonly used to facilitate design, evaluation, and implementation of evidence-based interventions,^35,36^ whereas the TDF is a conceptual model developed to identify influences on individuals and evaluate barriers and facilitators to behavior change.^37,38^ Their combined use, which allows question adaptation from all 5 CFIR domains (intervention characteristics, outer setting, inner setting, characteristics of the individuals involved, and process of implementation) and supplementation with TDF constructs not otherwise captured (eg, those relating to an individual’s professional role),^37,39^ has previously been applied in pediatric and primary care settings to design, evaluate, and implement interventions.^40,41,42^ The research team contacted the identified stakeholders from the 4 participating hospitals, and 2 authors trained in qualitative analysis (J.M.W. and M.C.) conducted semistructured interviews via videoconference from May to July 2021.^43^ Interview probes were introduced and tailored to the expertise of each stakeholder. All interviews were audio-recorded and transcribed verbatim.

Transcript data were analyzed iteratively through a combined inductive and deductive coding approach using NVivo, version 12 (QSR International). An initial codebook was developed using deductive codes from CFIR and TDF domains. Several rounds of open coding identified inductive subcodes that represented themes in relation to this study. After a codebook was created, 2 authors (J.M.W. and M.C.) independently coded each transcript and met to resolve coding discrepancies and iteratively revise the codebook and code definitions as further interviews were conducted.^44,45^ Interviews were conducted until data saturation was achieved for the final code book, and Cohen κ was calculated to assess intercoder reliability (Cohen κ = 0.78; eAppendix 2 in Supplement 1).^46^ To facilitate cross-site analysis, coded transcript data were charted into a framework matrix with rows organized by site and columns by constructs.^47^ Thematic analysis of coded data was used to identify the specific determinants of TTS for patients with hip fracture across hospitals and place them into broader general theme areas.^48^ The resultant themes were discussed and refined (J.M.W. and M.C.), then critiqued by an experienced qualitative investigator (R.N.K.) to expand understanding of the data and ensure qualitative rigor.

### Sequential Mixed-Methods and Quantitative Assessment

The interviews and assessment were integrated (1) by building the initial assessment from the interview guide and (2) by incorporating emergent themes from the interviews into the final assessment. The contextual determinants for TTS identified from the qualitative study informed the development of the quantitative assessment to examine the frequency with which each potential determinant was cited by respondents as being important at their institution.^33,34^ The assessment included items regarding the presence of determinants within each theme (30 items) as well as items evaluating current pathways for hip fracture care (eg, mean number of geriatric hip fractures; 10 items). Participants were sent a link to the online survey via Qualtrics. All responses were kept confidential. Participant demographics and hospital characteristics from the American Hospital Association annual survey were also collected (eAppendix 3 in Supplement 1). Race and ethnicity data were collected because these characteristics may shape personal experiences and perspectives. Race and ethnicity were self-reported (American Indian or Alaska Native, Asian, Black or African American, Native Hawaiian or Other Pacific Islander, White, or other) and included as demographic characteristics. The other category includes other race (if no specific category fit), declined to answer, and prefer not to say or not specified. Responses were recorded from May to July 2022.

### Data Analysis

Descriptive statistics were calculated for demographic variables and individual items of the assessment. Comparisons of the barriers and facilitators across different hospital sites were made through calculating percentage agreement (proportion of shared determinants) within like characteristics (eg, all those within the same hospital setting) to determine the key factors that may affect TTS across diverse contexts. Data analysis was performed in August 2022, using Microsoft Excel Office 365, version 2207 (Microsoft Corp).

## Results

### Qualitative Analysis

Among the 43 stakeholders contacted, 34 (79%) participated in semistructured interviews. A total of 19 were men (56%) and 15 were women (44%). Reasons for nonparticipation included inability to schedule an interview (n = 3), nonresponse to email inquiry (n = 3), and preference to not participate (n = 3). The mean (SD) interview duration was 28 (9) minutes and ranged from 13 to 54 minutes. Interviewees included 13 orthopedic surgeons (38%; including 4 residents), 3 emergency medicine physicians (9%), 6 hospitalists (18%), 6 anesthesiologists (18%), 3 nurses (9%), and 3 clinical or support staff (9%). One interviewee held positions in both quality management and clinical care (Table 1). Participants represented hospitals of varying payer types (public vs private, government vs nongovernment), size (167-731 beds), trauma level (nontrauma ACS level I), teaching status, and preoperative care structure (comanagement vs orthopedic consult; eTable 1 in Supplement 1).

**Table 1.** Qualitative Study Participant Demographics

Demographic	No. of participants (%) (N = 34)
Staff position	
Orthopedic surgeon, attending	9 (26)
Orthopedic surgeon, resident	4 (12)
Emergency medicine physician	3 (9)
Hospitalist	6 (18)
Anesthesiologist	6 (18)
Operating room charge nurse	2 (6)
Orthopedic nurse navigator	1 (3)
Quality management staff^a^	1 (3)
Other clinical or support staff	3 (9)
Sex	
Male	19 (56)
Female	15 (44)
Study hospital	
A only	6 (18)
B only	7 (21)
C only	10 (29)
D only	7 (21)
Multiple^b^	4 (12)

^a^ One interviewee held positions in both quality management and clinical care.

^b^ Orthopedic surgery residents had rotations across multiple hospitals.

Four themes representing key determinants of TTS were identified from the interviews: availability, care coordination, improvement climate, and incentive structure. Representative quotations for each theme are illustrated in Table 2.

**Table 2.** Themes and Quotes From Qualitative Interviews

Theme and relevant code	Representative quote
**Availability**	
Staff availability	“The manpower is always the limiting issue on the weekend. There’s plenty of ORs, it’s just weekend staffing, both from an anesthesia standpoint and then also from an OR staff like OR nurses and surgical techs.”
Staff availability, pay structure	“We’re in private practice. We don’t have residents. People have to work the next day so they’re not going to be up at midnight or 2 am putting a hip back together. I don’t think anybody would find that a palatable goal.”
OR availability	“Wednesdays are horrible here—that’s our busiest day of the week, so if they come to us on that day with a hip fracture patient we’ll try and fit it in, but we can’t always get it that day.”
Weekend effect	“After hours and on weekends, let’s say from 7 pm to 7 am and on weekends, we’re down to one OR so there is that limitation.”
Weekend effect, case prioritization	“There’s very few ORs that are running over the weekend so other emergencies will usually bump us, because while like a hip fracture is urgent it’s not like something that again, you know we don’t like to do it in the middle of the night so we get bumped a lot and so you’re just kind of at the mercy of whatever comes in over the weekend.”
OR availability, weekend effect	“I would actually say that [Site D] is a great place to do surgery on the weekends, because we don’t have a trauma center so to speak, and we’re not doing liver transplants and stuff like that, so the OR is really pretty easy to book on the weekend. Generally speaking, we’re not fighting for OR time with other people. I’ve taken trauma call[s] both at [Site A] and at [Site D] and there’s absolutely no doubt it’s easier to get cases on weekends at [Site D] than at [Site A].”
**Care coordination**	
Communication, culture	“We’re [a] pretty small hospital, so we know, we’re on a first name basis with all the consultants. We have everybody’s cell phone number, especially when it’s something like cardiology it’s extraordinarily easy [to contact them].”
Preoperative clearance	“Occasionally the anesthesiologist will be a little bit more conservative than the hospitalist or the orthopedic surgeon and request cardiac clearance when neither of us had really felt that that was going to be necessary, and they see the patient last so if that happens it’s [*sic*] sometimes delays surgery. But to be honest, it is pretty uncommon. Partially because we’ve been burned enough in the past that we request cardiac clearance if we think that there’s even a whisper of a chance that it might be necessary.”
	“So, I think that sometimes they, in my opinion, do far too much in terms of preparatory work and not realizing that you know getting the hips back together in whatever way is deemed necessary and getting the patient back on their feet is something of a more urgent nature. So, my impression is they, in general, probably do way more than is necessary to get that patient into the operating room.”
Care pathway	“So, our protocol and the criteria specifically list that if the patient has a low risk or stable chronic medical condition that would benefit from comanagement with the medicine, those fall under a medicine co–follow-up. And then, if the patient has unstable or high-risk medical conditions then that is a primary medicine admission with ortho [co–follow-up].”
Preoperative testing, communication	“I think the number one mistake or error that happens in this pathway is inappropriate or incomplete workup of medical issues and less than perfect communication between services. That’s always where this breaks down, where it’s like we’re ready to take this person to the OR but either anesthesia or medicine hasn’t cleared them yet or medicine and anesthesia disagree on what the person needs before they go, but they are not talking to each other and we’re not appropriately putting everyone in touch and then that’s when delays happen.”
Care pathway, comanagement	“... the way that our team works is kind of a unique compared to a lot of places, where anyone on the team can actually order on a patient, as opposed to having a primary team and then consultants that basically just give advice and then depend on the primary team to order everything.”
	“Yeah, I think it’s actually quite good that everyone gets paged exactly the same moment. So, medicine, orthopedics, anesthesia because those are the three parties that need to be aware immediately that there’s a hip fracture patient in the ER.”
**Improvement climate**	
Time-consuming	“If you’re trying to take better care of a patient or be a better doctor for someone, then why wouldn’t you why wouldn’t you want to change, okay? The issues that you’ll run into are resources and workflow and workload. So, in general, things that make people’s jobs more difficult take more time or cause more stress and strain are not perceived as beneficial things when enacting change.”
Relative priority	“I’m not aware of any structured attempts to change the flow of how the hip fracture patients are managed. I’ve been around for many years, but I don’t recall if there’s been any attempt at that.”
Leadership	“You know, no nobody has ever really ever pushed that agenda very much in our arena, in terms of expediting surgery.”
Dissemination of information	“We have [a] conference every Wednesday where we review all the cases that happened the week prior and are upcoming for the week. It’s across all specialties so sports, joints, trauma, etc and so we often have discussions about what the latest research is and oftentimes it’s the residents who inform us, come with what’s been changing and then certainly our affiliation with [Site A] plays a big role in our you know, maintaining a more current understanding through grand rounds or weekly didactics, trauma conference.”
Individual stage of change	“... I’ve tried to like roll out pathways or programs in the past and unless people know why you’re doing whatever it is you choose to do, it doesn’t work.”
**Incentive structure**	
Elective case scheduling	“Often an issue is that [the ORs] are already filled up for the day and they don’t have an extra room to put the hip fracture in. They have a full elective schedule and that’s part of the economics of running a hospital. If you’re going to just leave a room in the OR open waiting for the hip fractures to show up, there’s going to be days where the ORs not going to be used and it won’t be very efficient, so it has to be looked at both from both sides.”
	“I don’t think any of the surgeons that take call there stop their so called ‘regular workflow’ that they already have scheduled when they take call.”
QI incentives	“There’s very close attention paid to quality metrics. We don’t have the same incentives as a lot of private sectors do but we spend a lot of time thinking about what good care is and then figuring out how to measure it and then figure out how to improve on it.”
Pay structure	“The compensation is just salary. I don’t get a dime if I come in on the weekend when I’m not on call. There’s no incentive to do that, other than just wanting to do the best thing for the patient.”
	“You know the ideal answer would be ‘Yeah, probably,’ but the reality is we don’t get paid to do that.”
Pay structure, staff availability	“We don’t pay as well as the other hospitals so it’s a big barrier for us to get nurses in. It’s very difficult.”

Abbreviations: ER, emergency room; OR, operating room; QI, quality improvement.

#### Availability

Across all sites, participants stated that timely access to resources (ie, for preoperative testing), adequate staffing, and open ORs were key determinants of TTS. Availability of ORs, staff, and surgeons varied depending on time and day of the week, with the degree of potential surgical delays and patterns of resource availability varying by site. A non–level I trauma center faced the challenge of having few, if any, trauma ORs available on the weekends, requiring hip fracture cases to occur between or after scheduled elective procedures, further delaying surgery. In contrast, level I trauma centers maintained a weekend trauma OR, but OR availability was dictated by the relative priority of cases, with variability in the perceived urgency of hip fracture cases. Other barriers related to availability included lack of staffing necessary to complete preoperative testing and treatment of patients.

#### Care Coordination

Participants emphasized that the interdisciplinary nature of hip fracture care requires effective coordination between stakeholders regarding initial diagnosis, admission, consultation, and medical evaluation. Steps of care requiring communication and consultation with additional staff members were reported as sources of delays when communication was limited or otherwise inefficient. At sites without a documented hip fracture protocol, coordination for each patient was conducted on a case-by-case basis and interviewees noted that efficiency relied on personal relationships to facilitate communication. However, unintentional delays occurred when the absence of clear guidelines led to uncertainty regarding the admitting service or the necessity of preoperative testing. At sites with a documented protocol, participants stated that the standardized steps of the preoperative workup reduced the burden on individual stakeholders and enabled them to focus on completing defined tasks within set time frames. For example, when a patient with hip fracture arrived at the emergency department, medicine, anesthesia, and orthopedics were simultaneously paged for evaluation and expected to respond within the hour. Protocols also included predetermined guidelines for choosing the admitting service and autopopulating order sets that minimize unnecessary preoperative testing.

#### Improvement Climate

The ability to implement changes to hip fracture care was highly dependent on the improvement climate, defined by the presence of a QI infrastructure, strong leadership, and structured avenues for implementing and monitoring changes. When a QI infrastructure was present, participants noted that physician leaders drove implementation, hip fracture protocol information was consistently disseminated, and there was frequent cross-department collaboration and engagement with recent research. However, some participants expressed that preoperative hip fracture care had not been identified as a space in which to drive QI. At sites without QI programs, improvement initiatives relied on individual efforts and success depended on others’ acceptance. Lack of universal support was often attributed to insufficient education on the importance of QI efforts. Although participants acknowledged the value of clinical data to inform improvement efforts, no site had a system to distribute real-time data (eg, mean TTS) to members of the care team.

#### Incentive Structure

The incentive structure of each site was seen as an indirect determinant of TTS, often influencing staff availability, resources dedicated to implementation, and individual agency of stakeholders. Participants reported that compensation models particularly impacted surgeon availability, identifying a salaried pay structure as one that facilitates shared “ownership of patients.” This contrasted with private practice models that did not facilitate shared ownership of patients and did not incentivize surgeons to expedite an unanticipated hip fracture case over an elective case. Additionally, the heterogenous on-call panel, with surgeons from different practices or subspecialties, was identified as a large source of delay.

The presence of a QI program with financial incentives was reported as a key factor facilitating the development of a hip fracture protocol, as it provides financial compensation in addition to collaborative support from quality consultants. Despite acknowledging that expediting patients to surgery would likely result in improved patient outcomes, participants across several sites stated that in the absence of financial incentives, there was minimal motivation to spend additional time researching, implementing, and updating evidence-based protocols that were perceived to be beyond one’s role.

### Quantitative Analysis

Quantitative assessment items and corresponding qualitative codes and themes are included in eTable 2 in Supplement 1. A total of 23 participants completed the assessment, including 18 men (78%) and 5 women (22%). In terms of race, 2 respondents (9%) identified as Asian, 1 (4%) as Black or African American, and 20 (87%) as White; 2 (9) identified as being of Hispanic or Latino ethnicity. Participating hospitals had diverse representations of geography (located across 17 states), size (<300 to ≥1000 beds), surgical resources (<20 to >60 inpatient ORs), hospital type (eg, nonprofit, for profit, or government), financial structures (eg, integrated salary model, open physician-hospital organization, or closed physician-hospital organizations). Further baseline characteristics of respondents and their respective hospitals, including American Hospital Association characteristics, are listed in Table 3 and eTable 3 in Supplement 1. No sites shared identical profiles in characteristics and reported determinants of TTS (eFigure in Supplement 1). The mean (SD) percentage of shared determinants across sites was 56% (6%). Among sites with the same system characteristics (eg, setting), the percentage of shared determinants ranged from 52% to 81%; among sites with similar size characteristics (eg, number of beds), the percentage of shared determinants ranged from 52% to 60% (eTable 4 in Supplement 1).

**Table 3.** Quantitative Study Participant and Hospital Characteristics

Characteristic	Value (N = 23)
**Respondent, No. (%)**	
Race and ethnicity	
Asian	2 (9)
Black or African American	1 (4)
Hispanic or Latino^a^	2 (9)
White	20 (87)
Practice type	
Orthopedic trauma surgery	19 (83)
Subspecialty practice and treat hip fractures	3 (13)
General orthopedic surgery	1 (4)
Sex	
Male	18 (78)
Female	5 (22)
**Hospital and system, mean (range)**	
Patient race and ethnicity distribution, estimated %	
American Indian or Alaska Native	1 (0-15)
Asian	7 (0-30)
Black or African American	20 (2-60)
Hispanic or Latino	16 (5-44)
Native Hawaiian or Other Pacific Islander	1 (0-20)
White	20 (10-90)
Multiple	3 (0-10)
Other^b^	10 (0-44)
Patient insurance, estimated %^c^	
Health maintenance organization	21 (0-50)
Medicare	51 (0-93)
Medicaid	10 (0-30)
Uninsured	10 (0-39)
Military	1 (0-5)
Other	8 (0-100)
**Hip fracture pathway, No. (%)**	
Mean monthly geriatric hip fracture volume^c^	
≤10	10 (44)
11-20	8 (35)
>20	4 (17)
Teaching status	
Teaching	20 (87)
Nonteaching	3 (13)
Hospital setting	
Urban	16 (70)
Suburban	5 (22)
Rural	2 (9)
No. of dedicated weekday orthopedic trauma rooms (9 am to 5 pm)	
0	5 (22)
1-2	11 (48)
2-3	6 (26)
4-5	1 (4)
No. of dedicated orthopedic trauma rooms after hours (5 pm to 7 am)	
0	20 (87)
1	2 (9)
2	1 (4)
Formal comanagement system	
Yes	18 (78)
No	5 (22)
Dedicated general operating room after hours	
Yes	22 (96)
No	1 (4)
Support for quality improvement projects	
Yes	21 (91)
No	2 (9)
Financial incentives for quality reporting metrics	
Yes	8 (35)
No	15 (65)

^a^ Hispanic or Latino ethnicity was calculated separately.

^b^ The other category includes other race (if no specific category fit), declined to answer, and prefer not to say or not specified.

^c^ Missing data.

Across the 30 contextual determinants surveyed, the most reported barriers to reducing TTS were lack of OR availability (11 [48%]), a soft protocol for coordination of care (12 [52%]), a lack of power to change (5 [22%]), and a lack of monetary incentive for urgent cases (9 [39%]) (Table 4). The most reported facilitators were available ORs (13 [57%]), a formal comanagement system (14 [61%]), the presence of a physician champion with a focus on urgent surgery for patients with hip fracture (18 [78%]), and the existence of programs supporting improvement work (11 [48%]). Further frequency data of determinants grouped by theme are available in Table 4.

**Table 4.** Frequency of Barriers and Facilitators to Reduced Time to Surgery

Theme^a^	No. of respondents (%)	
	Barrier	Facilitator
**Availability**		
OR (no or yes)	11 (48)	13 (57)
OR staff or anesthesia (no or yes)	10 (44)	9 (39)
Surgeon (no or yes)	3 (13)	10 (44)
Staff for testing (eg, echocardiogram) (no or yes)	9 (39)	7 (30)
Internal medicine or hospitalist (no or yes)	2 (9)	6 (26)
Prioritization or triage system (no or yes)	7 (30)	6 (26)
**Care coordination**		
Protocol for coordination and workup (soft or formal)	12 (52)	10 (43.5)
Distinct responsibilities of care team (no or yes)	3 (13)	11 (48)
Comanagement process (informal or formal)	4 (17)	14 (61)
**Improvement climate**		
Ability or power to change how hip fractures are managed (limited or strong)	5 (22)	12 (52)
Support for quality improvement projects (limited or strong)	4 (17)	11 (48)
Physician champion for urgent hip fracture surgery (not present or present)	4 (17)	18 (78)
Continuous education on topic of hip fractures (not present or present)	NA	9 (39)
Structured dissemination of information, such as case reviews (not present or present)	NA	8 (35)
**Incentive structure**		
Heterogeneity of on-call panel	8 (35)	NA
Physician payment structure does not incentivize urgent hip fracture surgery	9 (39)	NA
Salary model leading to faster availability of surgeon	NA	9 (39)
Program that facilitates improvement work	NA	11 (48)

Abbreviations: NA, not applicable; OR, operating room.

^a^ Determinants of time to surgery for patients with hip fracture are grouped by identified themes: availability, care coordination, improvement climate, and incentive structure.

Among the sites in an urban setting, 10 of 16 (63%) reported “strong support for quality improvement projects” as a facilitator to reduced TTS, compared with 11 of 23 (48%) overall. Both sites without hospitalists involved in hip fracture care reported “lack of distinct responsibilities” as a barrier to reducing TTS. Of the 18 sites with formal comanagement systems, “strong ability or power to change” and “distinct responsibilities” were reported as facilitators in 12 (67%) and 11 (61%), respectively. These factors were not reported by hospitals without formal comanagement. The majority of nonfederal government hospitals (4 of 5 [80%]) reported lack of OR staff as a barrier compared with fewer than one-third of private hospital sites (4 of 14 [29%]). All nonteaching hospitals reported lack of OR access as a barrier, compared with 8 of 20 teaching hospitals (40%). Nonteaching hospitals reported a median of 5 (range, 4-6) barriers, whereas teaching hospitals reported a median of 4 (range, 0-10) barriers.

In large hospitals (500-1000 beds), frequently cited facilitators included the presence of distinct responsibilities (10 of 15 [67%]) and formal comanagement (9 of 15 [60%]). These were not reported in the largest hospitals (>1000 beds) that had the greatest frequency of barriers due to limited power (3 of 3 [100%]) and lack of staff for the OR and testing (2 of 3 [67%]). Hospitals with dedicated weekday orthopedic trauma ORs reported a median of 7 (range, 1-16) facilitators, compared with a median of 6 (range, 0-9) facilitators by hospitals without dedicated weekday ORs. All 3 hospitals with dedicated weekend orthopedic ORs reported “continuous education” as a facilitator and none reported “heterogeneity of on-call panel” as a barrier compared with the 20 hospitals without dedicated weekend ORs, of which 6 (30%) reported the respective facilitator and 8 (40%) reported the respective barrier.

## Discussion

Delaying surgery for patients with hip fracture past 24 hours increases morbidity and mortality,^7,49,50,51,52^ and the underlying cause of these delays includes both patient- and facility-level factors.^10,53^ This study described 4 themes of contextual determinants of TTS (availability, care coordination, improvement climate, and incentive structure) and barriers and facilitators within these themes that were present across diverse hospitals.

Stakeholders frequently stated that hip fracture care requires effective coordination that can be facilitated by standardized protocols, a claim supported by frequencies of the coordination determinants among many sites. However, approximately half of the sites with formal comanagement programs did not identify comanagement as a facilitator, consistent with literature suggesting that these programs have various levels of success in reducing TTS.^25,26,54,55,56,57^ Given that these programs are developed to delineate clear responsibilities of medical management in the preoperative evaluation period,^56^ their success could rely on the time taken for patient comorbidity optimization.

Furthermore, programs are frequently more successful at achieving desired outcomes when multiple complementary strategies are used.^54,58^ For example, although a comanagement program may reduce burden on surgeons, it increases the burden for other stakeholders who may not have the resources necessary for the change. In such cases, strategies like network weaving and resource-sharing agreements have been reported to provide buy-in for other stakeholders,^58^ and they may address issues of availability identified as crucial determinants by all but one site. Stakeholders frequently suggested that the ability to implement changes to care was dependent on the improvement climate, and this view was shared across the collection of sites. A physician champion was the most frequently reported facilitator.

In this study, collected data did not include the mean TTS for patients with hip fractures at each hospital, and assumptions cannot be made about TTS based on the presence of a single characteristic because inconsistencies persist in the literature regarding what is favorable.^59,60^ The 2 sites that shared identical characteristics differed considerably in contextual determinants, sharing just 57% similarity. Furthermore, we observed that larger teaching hospitals with more surgical resources, hospitalists involved in care, and formal comanagement systems tended to report more facilitators than barriers, but one of the largest hospitals in our study reported fewer facilitators than barriers, reporting the most barriers overall. This teaching hospital did not have involved hospitalists, a formal comanagement system, or dedicated weekend ORs.

### Limitations

This study has some limitations. Because most perspectives came from clinicians and did not include hospital leadership (who would have additional perspectives on resource allocation) or patients and their caregivers, our results may be biased by our interviewees. In addition, although interviewees came from different hospitals, they shared the same geographical area. To address this limitation, national sites were used in the quantitative phase to assess the generalizability of our findings. Although we developed an interview guide to minimize leading questions, it is possible that interviewer bias may have affected the qualitative analysis. Because TTS was not recorded for these sites and our sample was limited, we were unable to analyze the statistical significance of correlations between determinants and TTS or site characteristics.

## Conclusions

In this mixed-methods qualitative study, no 2 hospitals shared the same determinant profile, which highlights the heterogeneity in causes of delays and emphasizes the need for tailored interventions based on contextual determinants. We previously reported that successful implementation efforts using evidence-based interventions should be designed around prospectively identified determinants within a given local setting to increase their efficacy.^58^ However, no framework exists for doing this in hip fracture care; therefore, a resource that matches identified site barriers to successful improvement programs is needed. A matrix using the CFIR to identify barriers and match them to implementation strategies from the Expert Recommendations for Implementing Change list^61^ is an example of a tool that could be designed to provide sites with specific and tailored steps. Such tools may provide a scalable way to address TTS for patients with hip fracture nationally.

## Notes

Supplement 1. **eTable 1.** Qualitative Study Hospital Characteristics **eTable 2.** Quantitative Survey Items With Sample Qualitative Open Codes and Corresponding Themes **eTable 3.** American Hospital Association Hospital Characteristics **eFigure.** Site Heterogeneity Matrix **eTable 4.** Site Similarities **eAppendix 1.** Qualitative Interview Guide **eAppendix 2.** Codebook/Open Codes **eAppendix 3.** Quantitative Assessment
eReferences

Supplement 2.
Data Sharing Statement
