# Supplementary material for: Contextual Determinants of Time to Surgery for Patients With Hip Fracture
Source: JAMA Netw Open. 2023 Dec 15;6(12):e2347834. doi: 10.1001/jamanetworkopen.2023.47834 (PMC10724766; doi:10.1001/jamanetworkopen.2023.47834)
Supplement: Supplement 1. — eTable 1. Qualitative Study Hospital Characteristics eTable 2. Quantitative Survey Items With Sample Qualitative Open Codes and Corresponding Themes eTable 3. American Hospital Association Hospital Characteristics eFigure. Site Heterogeneity Matrix eTable 4. Site Similarities eAppendix 1. Qualitative Interview Guide eAppendix 2. Codebook/Open Codes eAppendix 3. Quantitative Assessment eReferences [file jamanetwopen-e2347834-s001.pdf]

Supplementary Online Content

Welch JM, Gomez GI, Chatterjee M, et al. Contextual determinants of time to surgery for patients with hip fracture. *JAMA Netw Open*. 2023;6(12):e2347834. doi:10.1001/jamanetworkopen.2023.47834

eTable 1. Qualitative Study Hospital Characteristics

eTable 2. Quantitative Survey Items With Sample Qualitative Open Codes and Corresponding Themes

eTable 3. American Hospital Association Hospital Characteristics

eFigure. Site Heterogeneity Matrix

eTable 4. Site Similarities

eAppendix 1. Qualitative Interview Guide

eAppendix 2. Codebook/Open Codes

eAppendix 3. Quantitative Assessment

eReferences

This supplementary material has been provided by the authors to give readers additional information about their work.

© 2023 Welch JM et al. *JAMA Network Open.*

eTable 1. Qualitative Study Hospital Characteristics

ID	A	B	C	D
**Hospital type**	Academic private medical center	University affiliated private hospital	Public tertiary teaching and research hospital	Government teaching hospital
**Number of Beds**	613	167	731	642
**Trauma Designation**	Level 1 Trauma Center	Non-level 1 Trauma Center	Level 1 Trauma Center	Non-level 1 Trauma Center
**Teaching Status**	Teaching	Non-teaching	Teaching	Teaching
**Preoperative Care Structure**	Hospitalist/orthopaedic or medicine co-management via documented protocol	Hospitalist management, orthopaedic surgeon consult	Hospitalist/orthopaedic or medicine co-management via documented protocol	Hospitalist/orthopaedic or medicine co-management

© 2023 Welch JM et al. *JAMA Network Open.*

eTable 2. Quantitative Survey Items With Sample Qualitative Open Codes and Corresponding Themes

Open Code Examples	Qualitative Theme	Quantitative Survey Items
Staff/Surgeon availability<br>OR availability<br>Weekend effect<br>Case prioritization<br>Pay structure<br>Weekend staffing, Nurse staffing<br>Block OR schedule	Availability	1. Does your hospital have a dedicated orthopaedic trauma room during the weekday (7AM-5PM)?<br>2. Does your hospital have a dedicated orthopaedic trauma room for after-hours/on call emergencies (5PM-7AM)?<br>3. Does your hospital have a general OR room for after-hours/on call emergencies?<br>4. Please select which factors related to **Availability** impact time to surgery at your hospital<br>o (Lack or Presence of) OR availability<br>o (Lack or Presence of) Surgeon availability<br>o (Lack or Presence of) OR staff/anesthesia availability<br>o (Lack or Presence of) Internal medicine/hospitalist availability<br>o (Lack of or Presence of) Staff for testing (e.g., ECHO) availability<br>o (Lack of or Presence of) Prioritization/ triage system
Preoperative testing/clearance<br>Care pathway, absence of protocol<br>Co-management<br>Communication, collaboration across services<br>Organizational norms<br>Admitting service decision, surgical involvement in medical management	Care Coordination	1. Do hospitalists provide care for hip fracture patients in your hospital?<br>2. Does your hospital have a formal co-management system with internal medicine/hospitalist medicine to manage hip fracture patients?<br>3. Please select which factors related to **Care Coordination** impact time to surgery at your hospital<br>o (Soft or Formal) co-management process<br>o (Lack or Presence of) Distinct responsibilities of care team<br>o (Soft or Formal) protocol for coordination and workup
QI infrastructure, QI leadership, champion<br>Individual stage of change, attitude toward change<br>Time consuming, Relative Priority<br>Informal monitoring of TTS<br>Dissemination of information	Improvement Climate	1. Is there hospital/system support for department quality improvement efforts?<br>2. Are there hospital financial incentives for surgeons tied to quality reporting metrics?<br>3. Please select which factors related to **Improvement Climate** impact time to surgery at your hospital<br>o (Lack or Presence of) physician champion for urgent hip fracture surgery<br>o (Lack or Presence of) ability or power to change how hip fractures are managed<br>o (Lack or Presence of) support for quality improvement projects<br>o Continuous education on topic of hip fractures<br>o Structured dissemination of information, i.e., case reviews
QI incentives, monetary incentives<br>Pay structure, salary model<br>Elective case scheduling	Incentive Structure	1. Does the hospital/system participate in bundled payments for total joint arthroplasty?<br>2. With which of the following types of payers does your hospital have a bundled payment arrangement for total joint arthroplasty?

© 2023 Welch JM et al. *JAMA Network Open.*

		3. Does your hospital/system own or jointly own a /health plan?<br>4. Please select which barriers related to **Incentive Structure** impact time to surgery at your hospital<br>o Heterogeneity on call panel<br>o Physician payment structure does not incentivize urgent hip fracture surgery<br>5. Please select which facilitators related to **Incentive Structure** impact time to surgery at your hospital<br>o Program that facilitates improvement work<br>o Salary model leading to faster availability of surgeon

© 2023 Welch JM et al. *JAMA Network Open.*

eTable 3. American Hospital Association Hospital Characteristics

**Characteristic**		No. (%)
**Type of Hospital/System**		
	For-profit	3 (13%)
	Nongovernment, not-for-profit	14 (61%)
	Government, nonfederal	5 (22%)
	Government, federal	1 (4%)
**Number of Beds**		
	<300	4 (17%)
	300-599	1 (4%)
	600-999	15 (65%)
	1,000+	3 (13%)
**Inpatient ORs***		
	<20	6 (26%)
	20-39	9 (39%)
	40-59	3 (13%)
	60+	4 (17%)
**Translation Services**		
	Yes	22 (96%)
	No	1 (4%)
**Pain Management Program**		
	Yes	21 (91%)
	No	2 (9%)
**Does your hospital/system own or jointly own a /health plan?**		
	Yes	9 (39%)
	No	14 (61%)
**Do hospitalists provide care for hip fracture patients in your hospital?**		21 (91%)
**Trauma Center**		
	Level 1	20 (87%)
	Level 2	1 (4%)
	Level 3	1 (4%)
**Social Services**		23 (100%)
**Bundled Payment for Total Joint Arthroplasty?**		
	Yes	14 (61%)
	No	9 (39%)
**What type of bundled payment?***		
	Traditional Medicare	5 (36%)
	A commercial insurance plan including ACA participants, individual, group or employer markets	2 (14%)
	Both	5 (36%)
**Hospital Owned by Physician/Physician Group**		0 (0%)
**Financial Arrangements**		
	Independent Practice Association (IPA)	2 (9%)
	Group practice without walls	1 (4%)
	Open Physician-Hospital Organization (PHO)	1 (4%)
	Closed Physician Hospital Organization (PHO)	6 (26%)
	Management Service Organization (MSO)	0 (0%)
	Integrated Salary Model (arrangements under which hospitals salary physicians to provide medical care)	9 (39%)
	Equity model	0 (0%)
	Foundation	1 (4%)
	Other	3 (13%)

*Missing data

© 2023 Welch JM et al. *JAMA Network Open.*

eFigure. Site Heterogeneity Matrix. A visual depiction of unique characteristics and determinants at each site. Characteristics (A-B): Colors (white, blue, orange, etc.) represent categorical variables within characteristics (e.g., dichotomous variables are indicated by two colors). Determinants (C-F): Red signifies the presence of the specific barrier, green signifies the presence of the specific facilitator, white signifies no selection of the determinant as a barrier or facilitator, while black signifies selection of the determinant as both a barrier and facilitator. (A) System Characteristics: Teaching, Type, Setting, Payment Model, Hospitalist Involved in Care, Formal Co-management; (B) Site Characteristics: Number of Beds, Number of ORs, Dedicated Orthopaedic OR, Number of Orthopaedic ORs; (C) Determinants within Coordination: Formal Protocol, Distinct Roles, Formal Co-management; (D) Determinants within Improvement Climate: Strong Support, MD Champion, Power to Change, Continued Education, Structured Discussions; (E) Determinants within Availability: OR Staff, ORs, Surgeon; Prioritization, Testing, IM/Hospitalist; (F) Determinants within Incentive Structure: Heterogenous On-call panel, Physician payment model, Financial Program that Facilitates Improvement Work.

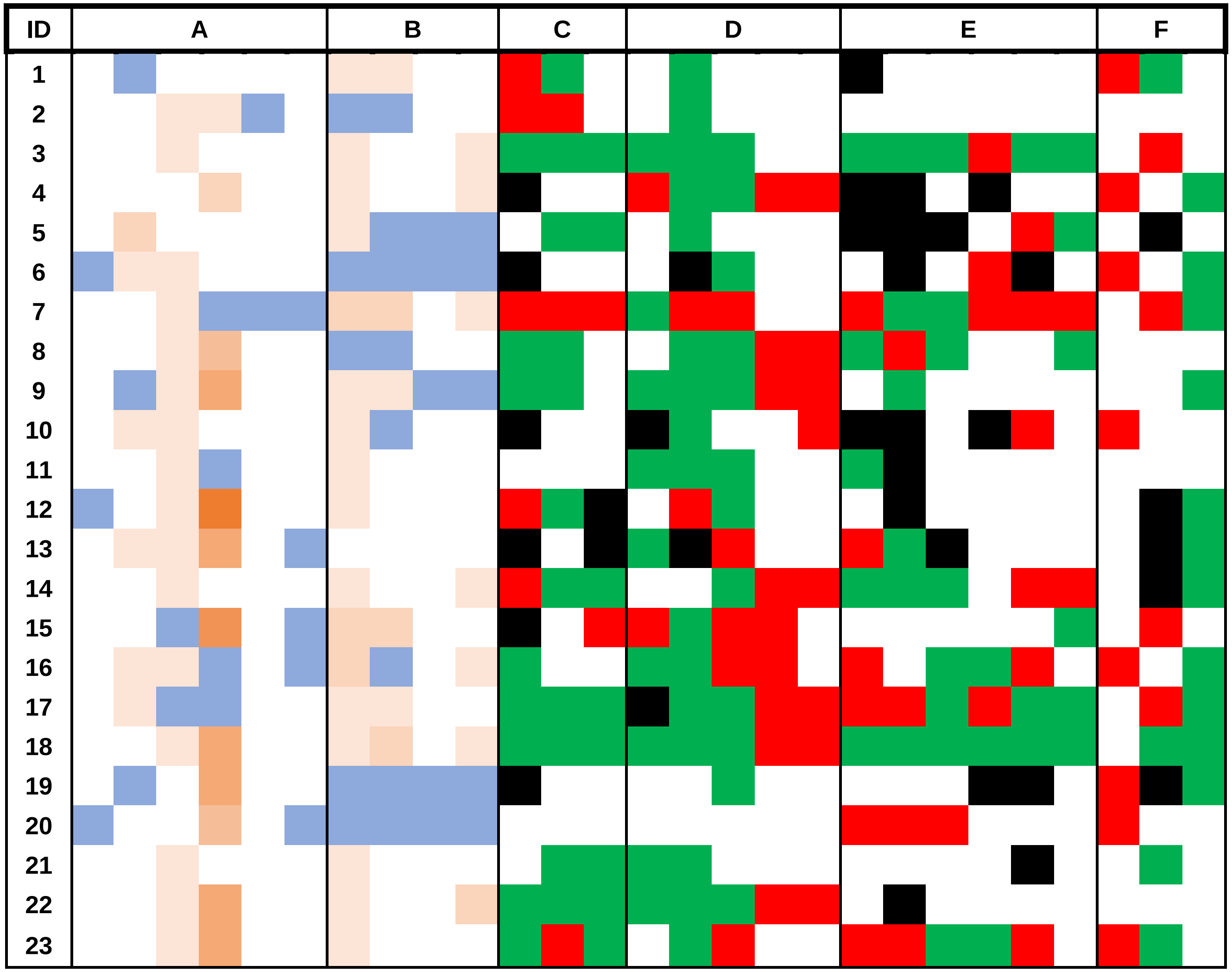

© 2023 Welch JM et al. *JAMA Network Open.*

eTable 4. Site Similarities

Characteristic Shared	Percent of Shared Characteristics and Determinants	Percent of Shared Determinants
**Teaching**, mean (SD)		
Yes	53.3 (9.8)	54.0 (10.6)
No	60.2 (7.5)	58.1 (5.6)
**Type**, mean (SD)		
For profit	48.8 (12.9)	45.2 (5.6)
Nongovernment, not for profit	51.6 (8.4)	53.8 (11.0)
Government, non-federal	64.6 (10.4)	57.4 (11.4)
Government, federal	-	-
**Setting**, mean (SD)		
Rural	54.0 (*)	58.0 (*)
Suburban	50.2 (7.8)	52.9 (9.6)
Urban	55.2 (10.2)	54.2 (10.5)
**Payment Model**, mean (SD)		
Other	73.2 (2.4)	55.9 (3.7)
Integrated Salary Model	55.6 (6.2)	56.5 (13)
Foundation	-	-
Open Physician-Hospital Organization (PHO)	-	-
Independent Practice Association (IPA)	54.0 (*)	81.0 (*)
Closed Physician Hospital Organization (PHO)	48.9 (9.9)	54.8 (10.1)
Closed Physician Hospital Organization, Integrated Salary Model	-	-
Unknown	-	-
**Hospitalist Involved in Hip Fracture Care**, mean (SD)		
Yes	54.0 (10.0)	54.3 (10.3)
No	49.0 (*)	51.6 (51.6)
**Formal Co-management System**, mean (SD)		
Yes	52.2 (9.7)	54.5 (10.8)
No	59 (9.5)	58.1 (9.6)
**Number of Beds**, mean (SD)		
<300	51.7 (8.3)	56.1 (12.5)
300-599	-	-
600-999	52.8 (10.0)	54.8 (11.4)
1000+	53.7 (6.0)	60.2 (1.9)

© 2023 Welch JM et al. *JAMA Network Open.*

Characteristic Shared (continued)	Percent of Shared Characteristics and Determinants (continued)	Percent of Shared Determinants (continued)
**Number of ORs**, mean (SD)		
<20	54.5 (9.8)	58.3 (10.3)
20-39	53.9 (7.3)	51.6 (10.7)
40-59	49.6 (17.6)	55.9 (6.7)
60+	48.0 (3.7)	60.2 (8.1)
**Dedicated Orthopaedic Trauma OR**, mean (SD)		
Yes	53.2 (9.6)	54.4 (10.2)
No	53.7 (7.7)	52.9 (8.2)
**Number of Dedicated Orthopaedic Trauma ORs**, mean (SD)		
0	53.7 (7.7)	52.9 (8.2)
1-2	53.7 (9.3)	55.3 (10.7)
2-3	48.8 (11.2)	54.8 (10.1)
4-5	-	-

(-) Calculations limited to sample size
(*) Standard deviation not applicable due to sample size

eAppendix 1. Qualitative Interview Guide

Introduction:

1. Can you describe your role in the care of a hip fracture patient at [** hospital]?
2. What kind of information or evidence are you aware of that is relevant to the time to surgery for hip fractures?
    a. (See what level of involvement they have, how much they have a desire to changes things, etc.)
    b. What do you know about --- (importance of timely surgery for hip fracture patients)?
3. What do you think is the optimal time frame for getting a person to surgery?
    a. Probe: Does that change based on the specific patient?

Topic 1: Care Pathway & main barriers

Structural Characteristics

1. What's the typical workflow for a hip fracture patient that is admitted during the day?
    - Probe: What about at night? What about on the weekend?
    - Probe: Are there any circumstances in which this workflow differs, such as on weekends or holiday?
2. How does the infrastructure or availability of consulting services (for example certain specialties or other personnel) affect time to surgery?
3. How to day-to-day conditions in the hospital impact time to surgery?
    - Does the need for coordination? Do challenges with coordination or availability of personnel limit the ability to get a patient to OR
    - Probe: Hospitalist service that sees patients on nights or weekends? Co-management of patients with internal medicine/geriatrics? Availability of teams for echoes (TTE)?
4. Are there specific resources, for example designated open OR time, for hip fractures?
    - How does the availability of resources affect time to surgery? Are there examples or times when resource constraints led to delayed surgery?
5. What kinds of changes to the infrastructure or services would need to occur to expediate patients to the OR?
    - How can the current workflow, for example you mentioned "**", be changed to reduce surgical delays?
    - Probe: Changes in formal policies? Changes in staffing?
    - What kind of approvals will be needed to introduce new interventions or alter existing infrastructure? Who will need to be involved?
    - Can you describe the process that will be needed to make these changes?
6. Are there any factors that lead to delayed time to surgery that would be more difficult to overcome?

Topic 2: Information/ background on TTS knowledge

Tension for Change

1. Is there a need to fix hip fractures urgently? Probe: within 24 hours? 48?
    - Why or why not?
2. Has there been any previous attempts to alter the workflow that currently exists?
    - What changed?
3. Is there anything that should NOT be changed about the process of care?

External Policies & Incentives

1. What kind of local, state, or national performance measures, policies, regulations, or guidelines influence the management of hip fractures?
    - Probe: Do you regularly discuss the publication of new guidelines with other staff members?
2. What kind of financial or other incentives influence management?
    - Could be specific to hip fractures or financial incentives in general

© 2023 Welch JM et al. *JAMA Network Open.*

- Not necessarily a priority question

3. How to members of the care team become aware of new research/literature?

Cosmopolitanism

1. What kind of information exchange do you have with other outside organizations regarding the management of hip fractures?
   - Probe (if unclear): This may include things like national meetings or research presentations.
   - How does information about new research, guidelines, or policies get disseminated throughout members of the care team?
   - Probe: List serves, national meetings, residents/fellows, registries

Peer Pressure

1. Can you tell me what you know about any other organizations that have implemented interventions to accelerate time to surgery for hip fractures?
   - Has this information influenced the process of managing hip fractures?'
   - Do you know of other organizations addressing time to surgery for hip fractures?
     1. Do you know of any specific interventions?

Cost

1. What costs would be incurred to expedite surgery for hip fractures?
   - Probe: considering supply, investment, opportunity cost

Topic 3: Target for Change

Culture

1. How would you describe the culture of your organization? Of your own setting or unit?
   - Do you feel like the culture of your organization is different from the hospital/overall organization? In what ways?
2. How do you think your organization's culture (general beliefs, values, assumptions that people embrace) influence time to surgery for hip fractures?
   - Can you describe an example that highlights this?
   - Probes: Specific interpersonal relationships
3. Can you describe how mistakes are viewed within your organization?
   - Is the organization punitive? Are they shared, accepted, or used as learning moments?

Learning Climate

1. To what extent do you feel like you can try new things to improve your work processes?
   - Do you feel like you have the time and energy to think about ways to improve things?
   - Did you feel valued/respected by your supervisor for the role you played?
   - What role did your supervisor (or other leaders) play? What actions did they take?

Implementation Climate

1. How easily does your organization embrace changes towards improvement of quality of care?
   - Why?
2. Have other changes in the hospital been successfully implemented?
   - What made them successful/unsuccessful?
3. What are the challenges that come with trying to introduce quality improvement initiatives in your setting?
4. If there was a policy or initiative that required [**example?] do you think your behavior would change?
   - Would the behavior of your coworkers change?

© 2023 Welch JM et al. *JAMA Network Open.*

Compatibility

1. How would an intervention to expedite time to surgery for hip fractures fit with existing work processes and practices in your setting?
    - What are likely issues or complications that may arise?
2. How would an intervention to expedite time to surgery be received in your workplace?

Relative Priority

1. How will an intervention to urgently fix hip fractures conflict with current organizational priorities?
    1. Would people value it? Do you seen any potential conflicts with implementation?
2. How complicated would it be to institute a process for hip fractures to be fixed within 24 hours of admission?
    1. Please consider the following aspects of the intervention: duration, scope, intricacy and number of steps involved and whether the intervention reflects a clear departure from previous practices.
    2. What do you think could most readily be changed in pursuit of 24 hours as a goal?

Organizational Incentives & Rewards

1. Are there any incentives or reward programs for quality improvement initiatives at your workplace?
    - Are there any related to hip fracture treatment?
    - Can you describe them?

Goals & Feedback

1. How are organizational goals for quality of care monitored for progress?
    - Can you give an example of monitoring in terms of the type of information, who is informed, and how?
2. What type of formal feedback do you receive about your work?
    - What do they look like? Content, mode, form?

Engaging

1. Who are the key individuals engaged in efforts to optimize time to surgery for hip fractures?
    - Are there people who are already motivated/making changes/getting others on board?

Opinion Leaders

1. Who are the key influential individuals to get on board with an intervention to reduce the time to surgery for hip fractures at your hospital?
    - Probe: Are there formal appointed implementation leaders? Are there QI positions?

Champions

1. Other than the formal implementation leader(s), are there people in your organization who are likely to champion (go above and beyond what might be expected) to create a change in time to surgery?
    - How can individuals be encouraged/engaged in trying to reduce time to surgery?
    - If you were to design an implementation, who would be key leaders to enforce/integrate this new intervention?

Topic 4: Individual Beliefs

Self-efficacy

1. What role do you play in time to surgery for hip fractures? What role does the orthopaedic surgeon play in time to surgery for hip fractures?

© 2023 Welch JM et al. *JAMA Network Open.*

2. Do you feel that it is part of your assigned role/job to ensure a patient gets to surgery in a timely fashion?
3. How confident are you that you will be able to reduce time to surgery for hip fracture patients?
    - What gives you that level of confidence (or lack of confidence)?
4. How confident do you think your colleagues feel?

Emotions

1. We know that clinician's emotions can affect their practice.
    - Do negative emotions such as frustration stress, or nervousness ever arise?
    - Do positive emotions such as satisfaction, relief, or hope arise?
2. Does your emotional state at the time (mood, feelings toward the patient, fatigue) influence your behaviors?
3. Do you think the emotion of other clinicians influence patient outcomes?

Individual Stage of Change[1,2]

1. How prepared are you to facilitate urgent surgery for hip fractures?
    - Knowledge stage (Precontemplation) - knowledge of key aspects of the intervention
    - Persuasion stage (Contemplation) - likes the intervention, discusses it with others, buys into it, has a positive view
    - Decision stage (Preparation) - intends to seek additional information and try it
    - Implementation stage (Action) - acquires additional information, uses intervention regularly, and has continued use
    - Confirmation stage (Maintenance) - recognizes benefits, has integrated the intervention into routines, promotes use to others.

© 2023 Welch JM et al. *JAMA Network Open.*

eAppendix 2. Codebook/Open Codes

CFIR Domain	Axial Code	Open Codes
Outer setting	Patient Factors	Patient mental status, comorbidities, mechanism of injury, coagulation status, age, needs translator
	Family/Social Context	Family decision making, patient support, family contact advocate
	National Guidelines	National guidelines, large databases, NSQIP, awareness of recent research
	Influence/Knowledge of Outside Organizations	Collaboration with teaching hospital, national trauma conferences
Inner Setting	Coordination	Medical decision making, echo, anesthesia consult, paging system, collaboration across services, admission decisions, communication, absence of protocol, co-management, multidisciplinary ordering, predefined roles
	Dissemination of Information	Internal emails, education for employees, journal clubs/discussion, cross department literature, conferences, CME, interdisciplinary meetings
	Measurement	No bandwidth to document, monitor pathway efficiency, infrastructure for data measurement, long term outcomes, quality of care, informal monitoring of TTS
	Culture	interpersonal relationships, organizational norms, expectations, patient centered values, goal oriented, feedback
	Availability	OR availability, Block OR schedule, surgeon availability, case prioritization,
	Implementation Climate	Presence/lack of QI infrastructure, time consuming, QI environment, mindset to improve, lack of QI specific leadership, data for QI, champion, priority level
	Preop Clearance	Anesthesiology consults, medical clearance, admitting service decision, surgical involvement in medical management
	Weekend Effect	Weekend OR, Weekend staffing, admission time, available trauma OR
	Employment/Pay Structure	Private practice, shift work, monetary incentives, staff availability, elective cases, nurse staffing
Characteristics of Individuals	Self-efficacy	Individual autonomy, confidence level of provider, individual desire to change
	Perceived efficacy of others	Leadership, responsibility to change, taking initiative
	Knowledge	Attitude toward change, knowledge of interventions, knowledge of guidelines/QM, familiarity with guidelines

© 2023 Welch JM et al. *JAMA Network Open.*

CFIR Domain (continued)	Axial Code (continued)	Open Codes (continued)
Characteristics of Individuals	Beliefs	Belief about care pathway, belief about need for intervention, belief about potential successes/failure, potential solutions
Process	Where to change next	Solutions, barriers
	Who leads or would lead a change	Leaders, champions, formally appointed leaders, coordinators
Current Care Pathway	Site A	Site A care pathway components
	Site B	Site B care pathway components
	Site C	Site C care pathway components
	Site D	Site A care pathway components

© 2023 Welch JM et al. *JAMA Network Open.*

eAppendix 3. Quantitative Assessment

Block 1
Name of person completing survey

Hospital/System Name

1. What is your type of orthopaedic surgery practice?
   - Orthopaedic trauma surgery
   - General orthopaedic surgery
   - Subspecialty practice and treat hip fractures

2. What is your race/ethnicity? Select all that apply
   - White
   - Black or African American
   - American Indian or Alaska Native
   - Asian
   - Native Hawaiian or Pacific Islander
   - Other

3. Are you Hispanic/ Latino?
   - Yes
   - No

4. To which gender identity do you most identify?
   - Male
   - Female
   - Prefer not to say
   - Other:

5. Please provide the estimated patient insurance composition of hip fracture patients by percent. The total must sum to 100.
   - Private (i.e., HMO, PPO)
   - Medicare
   - Medicaid
   - Self-pay / Uninsured
   - Military (i.e., VA, TriCare)
   - Other

6. On average, how many geriatric hip fractures does your hospital treat per month?

7. Is your hospital a teaching hospital?
   - Yes
   - No

8. What setting is your hospital located in?
   - Urban
   - Suburban
   - Rural

9. Please provide your hospital's estimated patient demographic composition by percent. The total must sum to 100.
   - White
   - Black or African American
   - American Indian or Alaska Native
   - Asian
   - Native Hawaiian or Pacific Islander

- Other
- Two or more races

10. Please estimate what percent of your hospital's patients are Hispanic/Latino.

American Hospital Association 2021 questions

1. Please indicate the type of hospital you work at
    - Government, non-federal (e.g., state or county)
    - Government, federal (e.g., VA)
    - Nongovernment, not for profit
    - For Profit

2. Is your hospital owned in whole or in part by physicians or a physician group?

3. How many beds are in your hospital?

4. How many inpatient operating rooms are in your hospital?

5. Do you have an on-campus emergency department?
    - Yes
    - No

6. Do you have linguistic/translation services?
    - Yes
    - No

7. Do you have a pain management program?
    - Yes
    - No

8. Do you have social work services?
    - Yes
    - No

9. Please indicate the type of financial arrangements you have with your hospital
    - Independent Practice Association (IPA) (Association of independent physicians that contract with independent care delivery organizations for a negotiated fee for service)
    - Group practice without walls
    - Open Physician-Hospital Organization (PHO)
    - Closed Physician Hospital Organization (PHO)
    - Management Service Organization (MSO)
    - Integrated Salary Model (arrangements under which hospitals salary physicians to provide medical care)
    - Equity model
    - Foundation
    - Other, please specify:

Hospital characteristics

1. Does your hospital have a dedicated orthopaedic trauma room during the weekday (7AM-5PM)?
    - Yes, how many?
    - No

2. Does your hospital have a dedicated orthopaedic trauma room for after-hours/on call emergencies (5PM-7AM)?
    - Yes, how many?
    - No

© 2023 Welch JM et al. *JAMA Network Open.*

3. Does your hospital have a general OR room for after-hours/on call emergencies?
- Yes
- No

4. Does the hospital/system participate in bundled payments for total joint arthroplasty?
- Yes
- No

5. With which of the following types of payers does your hospital have a bundled payment arrangement for total joint arthroplasty? (Check all that apply)
- Traditional Medicare
- A commercial insurance plan including ACA participants, individual, group or employer markets

6. Does your hospital/system own or jointly own a /health plan?
- Yes
- No

7. Do hospitalists provide care for hip fracture patients in your hospital?
- Yes
- No

8. Does your hospital have a formal co-management system with internal medicine/hospitalist medicine to manage hip fracture patients?
- Yes
- No

9. Is there hospital/system support for department quality improvement efforts?
- Yes, examples:
- No

10. Are there hospital financial incentives for surgeons tied to quality reporting metrics?
- Yes, examples:
- No

Block 2

The following questions are related to factors that impact time to surgery for hip fractures. We have previously interviewed sites and identified the following factors (Care Coordination, Improvement Climate, Availability, Incentive Structure). Please review the list below and select the barriers and facilitators that impact the time to surgery at your hospital.

1. Please select which barriers related to **Care Coordination** impact time to surgery at your hospital
- Lack of formal co-management process
- Lack of distinct responsibilities of care team
- Soft protocol for coordination and workup

2. Please select which facilitators related to **Care Coordination** impact time to surgery at your hospital
- Formal co-management process
- Distinct responsibilities of care team
- Formal protocol for coordination and workup

3. Please select which barriers related to **Improvement Climate** impact time to surgery at your hospital
- No strong physician champion for urgent hip fracture surgery
- No ability or power to change how hip fractures are managed
- Limited or no support for quality improvement projects

4. Please select which facilitators related to **Improvement Climate** impact time to surgery at your hospital

© 2023 Welch JM et al. *JAMA Network Open.*

- Strong physician champion for urgent hip fracture surgery
- Strong ability or power to change how hip fractures are managed
- Strong support for quality improvement projects
- Continuous education on topic of hip fractures
- Structured dissemination of information, i.e., case reviews

5. Please select which barriers related to **Availability** impact time to surgery at your hospital
    - Lack of OR availability
    - Lack of surgeon availability
    - Lack of OR staff/anesthesia availability
    - Lack of internal medicine/hospitalist availability
    - Lack of staff for testing (e.g., ECHO) availability
    - Prioritization/ triage system

6. Please select which facilitators related to **Availability** impact time to surgery at your hospital
    - OR availability
    - Surgeon availability
    - OR staff/anesthesia availability
    - Internal medicine/hospitalist availability
    - Staff for testing (e.g., ECHO) availability
    - Prioritization/ triage system

7. Please select which barriers related to **Incentive Structure** impact time to surgery at your hospital
    - Heterogeneity on call panel
    - Physician payment structure does not incentivize urgent hip fracture surgery

8. Please select which facilitators related to **Incentive Structure** impact time to surgery at your hospital
    - Program that facilitates improvement work
    - Salary model leading to faster availability of surgeon

© 2023 Welch JM et al. *JAMA Network Open.*

eReferences

1. Prochaska JO, Norcross JC. Stages of change. *Psychotherapy: Theory, Research, Practice, Training*. 2001;38(4):443-448. doi:10.1037/0033-3204.38.4.443

2. Rogers CR. A process conception of psychotherapy. *American Psychologist*. 1958;13(4):142-149. doi:10.1037/h0042129

© 2023 Welch JM et al. *JAMA Network Open.*
