# Supplementary material for: Contextual Determinants of Time to Surgery for Patients With Hip Fracture
Source: JAMA Netw Open. 2023 Dec 15;6(12):e2347834. doi: 10.1001/jamanetworkopen.2023.47834 (PMC10724766; doi:10.1001/jamanetworkopen.2023.47834)
Supplement: Supplement 2. — Data Sharing Statement [file jamanetwopen-e2347834-s002.pdf]

Data Sharing Statement

Welch. Contextual Determinants of Time to Surgery for Patients With Hip Fracture. *JAMA Netw Open*. Published December 15, 2023. doi:10.1001/jamanetworkopen.2023.47834

Data
Data available: No
